# Coastal Shorebirds Delay Maturity More Than Inland Ones

**DOI:** 10.1002/ece3.71679

**Published:** 2025-06-27

**Authors:** Danny I. Rogers, Theunis Piersma, Clive D. T. Minton, Adrian N. Boyle, Chris J. Hassell, Ken G. Rogers, Andrew Silcocks, Jorge S. Gutiérrez

**Affiliations:** ^1^ Arthur Rylah Institute for Environmental Research, Department of Energy Environment and Climate Action Heidelberg Victoria Australia; ^2^ Victorian Wader Study Group Melbourne Victoria Australia; ^3^ Australasian Wader Studies Group Melbourne Australia; ^4^ BirdEyes, Centre for Global Ecological Change at the Faculties of Science & Engineering and Campus Fryslân University of Groningen Leeuwarden the Netherlands; ^5^ Department of Coastal Systems NIOZ Royal Netherlands Institute for Sea Research Den Burg, Texel the Netherlands; ^6^ Rudi Drent Chair in Global Flyway Ecology, Groningen Institute for Evolutionary Life Sciences (GELIFES) University of Groningen Groningen the Netherlands; ^7^ Global Flyway Network Broome West Australia Australia; ^8^ Birdlife Australia Carlton Victoria Australia; ^9^ Department of Anatomy, Cell Biology and Zoology, Faculty of Sciences University of Extremadura Badajoz Spain; ^10^ Ecology in the Anthropocene, Associated Unit CSIC‐UEX, Faculty of Sciences University of Extremadura Badajoz Spain

**Keywords:** Charadrii, comparative biology, demography, life‐history strategies, life‐table variable, migration, over‐summering, waders

## Abstract

Delaying the age of first breeding will lower lifetime reproductive output unless compensated for by increased fecundity or survival. Yet, in many migratory shorebird species (Charadriiformes) individuals delay their first return migration to the breeding grounds until they are several years old. Using data from non‐breeding and breeding season counts of shorebirds in the non‐breeding areas, recaptures, and long‐term banding studies, we assess the age of first return migration (as a measure of maturity) for 37 shorebird species that have migrated to Australian non‐breeding grounds. We provide a comparative analysis of the association between the measure of maturity and habitat use during the non‐breeding period, contrasting coastal and inland wetland habitats. After controlling for latitudinal and phylogenetic covariates, we found a positive relationship between body size and the age of first return migration. However, there was still a stronger relationship with the type of non‐breeding habitat used. Coastal shorebird species delayed maturity more than species that spend the non‐breeding season in non‐tidal inland wetlands. This finding expands on previously identified ecological and physiological differences between coastal and inland shorebirds and leads to questions on the environmental characteristics embodied in the habitat contrast. We propose that the complicated tidal dynamics and differences in prey make it more difficult to become an individually competent coastal (rather than inland freshwater) forager.

## Graphical abstract

Migratory shorebirds frequenting coastal non‐breeding habitats delay maturity, as expressed in the age of first return migration, more than the species using inland freshwater habitats.

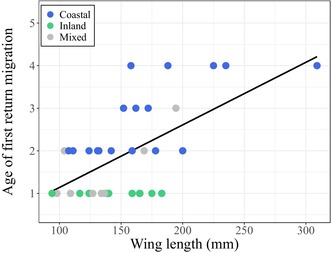

## 1 Introduction

Annual fecundity, annual adult mortality, pre‐reproductive survival, and the age of maturity determine the growth rate of animal populations (Caswell 1989; Oli and Dobson 2003; Stahl and Oli 2006). Some of these life‐table variables (notably fecundity) have been in focus in the long history of studies of avian life histories, with both theory and empirical evidence supporting some important basic patterns (Ricklefs 2000; Bennett and Owens 2002): (1) annual fecundity is directly proportional to annual adult mortality; (2) average annual pre‐reproductive mortality is directly related to annual adult mortality; and (3) age at maturity decreases with increasing annual mortality. As the life‐table variables interact so strongly, it is of great interest to understand the factors that influence them (Sæther and Bakke 2000), especially as the number of bird species known to be threatened continues to increase and conservationists seek ways to arrest or reverse population declines. Shorebirds (also referred to as waders) are often a focus of such attention, as they are of conservation concern worldwide (MacKinnon et al. 2012; IUCN 2025), with a disproportionate number of migratory species listed as threatened (Koleček et al. 2021).

Shorebirds are also a favorite group for comparative biological studies. They are all within the order Charadriiformes and have many morphological and ecological similarities, most being rather long‐legged, long‐winged, ground‐nesting species that habitually wade when foraging on shorelines or in shallow water. Nevertheless, they show great variation in (1) migration distances and staging strategies (Piersma 1987; Conklin et al. 2017), (2) the ecological nature of their wetland non‐breeding habitats (Piersma 2003), and (3) their mating systems (Pitelka et al. 1974; Reynolds and Székely 1997; Reneerkens et al. 2014). Shorebirds have a clutch size capped at four eggs, so interspecific variation in fecundity is small; population size and trajectory are therefore expected to be determined by the balance between the remaining life‐table variables. Of these, the age of breeding maturity provides an intriguing life‐table variable (Stahl and Oli 2006). Indeed, shorebird biologists have long debated the underlying behavioral and ecological causes of some shorebirds delaying their first return migration to the breeding grounds until they are at least 2 years old (Loftin 1962; McNeil et al. 1994; Summers et al. 1995; Hockey et al. 1998; O'Hara et al. 2005; Ydenberg and Hemerik 2024).

Piersma (1997, 2003, 2007) drew attention to the several interesting differences between the life‐history characteristics of migratory shorebirds inhabiting coastal (marine) versus inland (freshwater) habitats. Coastal shorebirds tend to have: (1) more restricted non‐breeding areas, as coastal non‐breeding areas are essentially one‐dimensional (Piersma 2003); (2) generally longer seasonal migrations (Piersma 1997); (3) lower exposure to some parasites, as blood parasite densities are lowest in shorebirds from coastal habitats (Mendes et al. 2005; Yohannes et al. 2009; Clark et al. 2016; Soares et al. 2016); (4) smaller population sizes, and hence greater potential for population bottlenecking and loss of genetic variation (Piersma 2003); (5) higher metabolic rates (Gutiérrez et al. 2012), and (6) differences in the expression of immunocompetence (Abad‐ Gómez et al. 2020). As basal metabolic rates indicate the metabolic investments necessary to maintain high energy expenditure overall (Kersten and Piersma 1987), life in coastal habitats apparently increases levels of energy expenditure in connection with tidally‐induced food restrictions, high salinity, and windy microclimates (Gutiérrez et al. 2011, 2012; Gutiérrez 2014). This could partly explain why freshwater species appear to have a lower ‘lifespan energy potential’ (spend less energy per maximum lifetime) than marine species (Goede 1993). Piersma (2003) suggested that these differences may enforce each other with implications for both population dynamics and evolution (including rates of subspeciation, Kraaijeveld 2008), but the association with life‐table variables remains to be established.

Here we investigate whether one such life‐table variable differs between coastal and inland shorebird species. Noting that survival estimates, apart from the many challenges to estimate them properly (e.g., Rakhimberdiev et al. 2022), vary greatly within and among species (Méndez et al. 2018; Scholer et al. 2020), we here focus on the age of maturity. Life‐history theory predicts that age of maturity will have a strong effect on population growth rate, especially in species with short generation times (Stahl and Oli 2006). It is expected to be correlated with annual adult survival (which in turn is usually correlated with pre‐breeding survival; Ricklefs 2000; Bennett and Owens 2002). The life histories of migratory shorebirds have features that make them a particularly suitable group in which to examine the evolution of age at maturity. Studies of delayed maturity in sedentary species typically require extensive marking and resighting programs to distinguish ‘floaters’ from breeding adults (e.g., Newton 1998). However, it is quite an easy distinction to make in migratory shorebirds, as pre‐breeders typically remain on the non‐breeding grounds, separated by many hundreds or even thousands of kilometers from the nesting areas (Loftin 1962; McNeil et al. 1994; Summers et al. 1995). Moreover, juvenile shorebirds typically migrate on a broad front without precise knowledge of their non‐breeding destinations (Cresswell 2014), they tend to be less site‐faithful than adults (Lourenço et al. 2016), and non‐breeding sites typically hold birds from multiple breeding areas (Wymenga et al. 1990). It is therefore unlikely that the age of maturity is subject to the same strong local variation as apparent adult survival (Leyrer et al. 2012), although there may be some variation in age of maturity over broad regional scales (O'Hara et al. 2005).

Many shorebirds nest in remote Arctic regions where there have been few long‐term studies (Lappo et al. 2012; Chagnon‐Lafortune et al. 2024), and in many shorebird species, young individuals seldom return exactly to their birthplace to breed (Kentie et al. 2014). As a result, there are few direct measures of age of sexual maturity (when shorebirds are first capable of reproducing) or the age of breeding maturity (when shorebirds first successfully raise offspring). Instead, we use age of first return migration as a direct correlate of the age of both sexual maturity and breeding maturity. We consider this a reasonable assumption, as most migratory shorebirds nest at high latitudes in habitats very different from those used for the rest of their annual cycle (e.g., Piersma 2003). They therefore cannot attempt to breed or breed successfully until they have completed their return migration to the breeding grounds. Moreover, there is good evidence from tracking studies that those individuals that migrate from Australia to the breeding grounds do attempt to nest on arrival. Such studies are available for eastern curlew *Numenius madagascariensis* (Morrick et al. 2022), whimbrel *Numenius phaeopus* (Kuang et al. 2020), bar‐tailed godwit *Limosa lapponica* (Battley et al. 2012; Chan et al. 2022), great knot *Calidris tenuirostris* (Chan et al. 2019), red knot *Calidris canutus* (Piersma et al. 2021), ruddy turnstone (Minton et al. 2011; Gosbell et al. 2012), sanderling (Lisovski et al. 2016), curlew sandpiper *Calidris ferruginea* and red‐necked stint *Calidris ruficollis* (Lisovski et al. 2021).

Our study is based on migratory shorebirds that spend their non‐breeding season in Australia. With one exception (double‐banded plover *Charadrius bicinctus* , discussed in the Methods section), these species are long‐distance migrants that breed at high latitudes in the northern hemisphere during the boreal summer (~mid‐May to early August). Breeding ranges differ between species (e.g., Iwamura et al. 2013); for some species they include steppes or marshes of temperate northern Asia, for others they include Arctic tundra in Siberia or Alaska. In adult migratory shorebirds of the East Asian Australasian Flyway, southern migration (~August–October), and the return northwards migration through East Asian Australasian (~April–May) both take ~1–2 months (Marchant and Higgins 1993; Higgins and Davies 1996; Choi et al. 2016; Chan et al. 2019) and the remaining 6 months or more the of annual cycle (~October–March) are spent at the non‐breeding grounds such as our study sites in Australia. Immature migratory shorebirds carry out a slower southward migration to Australia, arriving from September to late November (Choi et al. 2016). As we show below, the age at which these immatures carry out their first return migration to the northern hemisphere varies markedly between species. Immatures of some species remain in Australia for two or more years before their first return migration to the northern hemisphere.

In this paper, we investigate whether the age of first return migration differs between coastal and inland shorebirds. We infer estimates of the age of first return migration from non‐breeding grounds in Australia using (1) long‐term banding datasets in which retraps and morphologically aged individuals provide information on the age structure of birds that skip return migration; (2) comparisons of Australian summer and winter counts. We then compile species‐specific summaries of ecological traits (non‐breeding habitat use, migration distance, breeding latitude, body size) and test these as predictors of the age of first return migration using phylogenetic generalized linear mixed models in a Bayesian framework. Given the fundamental dichotomies between coastal and inland species mentioned above, we predict coastal shorebirds to delay maturity more than inland ones.

## 2 Materials and Methods

### 2.1 Terminology

The habit of remaining on non‐breeding grounds instead of migrating to breeding areas has been termed ‘over‐summering’ (e.g., Loftin 1962; McNeil et al. 1994; Martínez‐Curci et al. 2020). This is potentially confusing in species that migrate between the northern and southern hemispheres. In the southern hemisphere, adult shorebirds are most numerous during the austral summer (~Dec–Feb) and the ‘over‐summering’ period corresponds to the austral winter (~June–August; Navedo and Ruiz 2020). Instead, we refer to ‘breeding periods’, when all adults intending to breed should be absent from Australia and ‘non‐breeding periods’, when most, or all, of these adults are present in Australia.

Annual schedules of migratory shorebirds differ from species to species, so we checked a dataset on the timing of migratory departures from north‐western Australia (Lane and Jessop 1985; Broome Bird Observatory and Australasian Wader Studies Group, unpubl. data) and banding databases for the dates of the latest departure from, and earliest return dates to, south‐eastern and north‐western Australia. These databases, along with the published literature (Marchant and Higgins 1993; Higgins and Davies 1996; Choi et al. 2016) indicated that we could treat the non‐breeding period as 1 October to 1 March for all species except the double‐banded plover. This species breeds in New Zealand during the austral spring and early summer (~September–January, Pierce 1999); we treated its non‐breeding season in Australia as 1 February to 15 August. We treated the breeding season as 15 May to 9 August for most species that breed in the northern hemisphere, but applied different cut‐off dates for a few early‐ and late‐migrating species (Table A1).

### 2.2 Age Structure

We used data from long‐term banding studies of migratory shorebirds to describe the age structure of shorebird populations in south‐eastern and north‐western Australia during the breeding period. We analyzed data collected between 1980 and 2005 (Table A2). Most south‐eastern Australian shorebirds were banded in Victoria, a smaller proportion in south‐eastern South Australia. Most shorebirds from north‐western Australia were captured in Roebuck Bay or at Eighty‐mile Beach, with smaller numbers from adjacent freshwater wetlands and the Port Hedland Saltworks.

The shorebirds captured were aged on site, using a combination of plumage and wing moult characteristics (Rogers et al. 2005). In general, individuals in Australia captured in their first breeding period can be aged reliably on the basis of recognizable retained juvenile feathers, especially in the remiges and inner greater secondary coverts. Adults captured at the same time of year (with full breeding plumage) are also typically distinctive. However, not all species are equally easy to age. In addition, in some species, shorebirds in their second or third breeding period were difficult to distinguish from one another, though they could be distinguished from younger immatures and adults. Accordingly, in species for which sample sizes allowed, we also examined Australian age structure during the breeding period solely on the basis of known‐age retraps, all of which were retrapped in the region where they had initially been captured. Retraps were treated as being of known age if they had been aged as first‐year individuals in the austral summer when originally captured, as aging criteria based on plumage and moult are well understood and easy to apply at this time of year (Rogers et al. 2005). In general, there was good correspondence between known ages of retraps and the plumage/moult‐based ages assigned by banders in the field (Table A3).

### 2.3 Austral Summer and Winter Comparisons

Extensive banding data were not available for all Australian shorebird species, and in the breeding period, it was of course impossible to catch species that do not delay migration and therefore never spend the breeding period in Australia. We expected the proportion of the non‐breeding population that remains in Australia for the breeding period to be proportional to the age of first return migration, with higher proportions occurring in species which delay return migration for several years; in such species, more age cohorts would be represented in the population that does not migrate to the breeding grounds. In practice, this proportion is likely to also be affected by other factors, such as annual fluctuations in breeding success (Minton et al. 2005) and wetland availability in inland Australia, which may influence the number of shorebirds using coastal sites (Alcorn et al. 1994; Nebel et al. 2008; Clemens et al. 2016). Subtle interpretations of the winter‐summer ratio are therefore difficult, but the ratio can nevertheless help to identify the age of first return migration, especially in those species in which delayed maturity does not occur.

We obtained data on winter‐summer ratios from two sources. Shorebirds at key Australian sites are counted annually (Birdlife Australia 2020) during the austral summer (usually in January) and austral winter (June or early July). We analyzed data collected between 1980 and 2005, when counts were carried out for the Population Monitoring Project of the Australasian Wader Studies Group. Count coverage has varied between regions and years, and annual variations occur in the breeding success of many species of migratory shorebird (Minton et al. 2005). We restricted analysis to paired counts when data were available from a given site in a non‐breeding period and in the following breeding period. This prevented our calculations of winter‐summer ratios from being biased by unequal search effort in winter and summer, but did not wholly remove the potential biases that could be caused by variations in annual breeding success, especially for sites from which data were only available for a few years.

Some shorebird species are rarely seen during shorebird counts because of cryptic behavior, or their regular use of non‐wetland habitats. We obtained data on the seasonal occurrence of these species from the Atlas projects of Birdlife Australia up to 2005. Based on the records of presence/absence data from sites throughout Australia collected by volunteers, we calculated reporting rates (number of records/number of surveys carried out at sites where at least one species of migratory shorebird was present) for breeding and non‐breeding periods.

### 2.4 Ecological and Life‐History Traits

We assembled species‐specific data on ecological and life‐history traits that may associate with age at maturity: non‐breeding habitat, migration distance, breeding latitude, and body size. Each species was classified into one of three categories of non‐breeding habitat in Australia: ‘inland’ for species typically occurring in freshwater or other inland habitats; ‘coastal’ for species typically restricted to coastal habitats; and ‘mixed’ for species that use both habitats; a similar categorization was used by Jackson et al. (2021). The mid‐point of the breeding range of each species (based on maps published in Hansen et al. 2016, 2022) was used to calculate breeding latitude (in degrees) and migration distance. Migration distance was expressed as the logarithm of the great circle distance from the breeding grounds to a point mid‐way between the north‐western and south‐eastern Australia, except for those species which only occur regularly in south‐eastern Australia (for which we treated Port Phillip Bay as the centre of the non‐breeding range) or north‐western Australia (for which we treated Roebuck Bay as the centre of the non‐breeding range). Finally, variation in the age of maturity is, to some extent, a product of the allometric relationship between body size and life‐table variables (Méndez et al. 2018); we thus used the median of average male and average female wing lengths (Marchant and Higgins 1993; Higgins and Davies 1996) as a measure of size. A simple phylogenetic mixed model indicated that the length of the wing chord was highly correlated with body mass (MCMCglmm: slope estimate = 0.29, 95% lower and upper credible intervals (CI) = 0.228–0.343). This model explained a large part of the variation in wing length in our data, with a marginal *R* ^2^ = 0.78 and a conditional *R* ^2^ = 0.85. It was therefore necessary to exclude wing length or body mass from our model to avoid overfitting and collinearity issues; we retained wing length, as it does not vary with nutrient store levels and is therefore considered a superior index of structural size (Piersma and Davidson 1991). Model results were much the same had we used body mass instead (see Figure 2a and Appendix Table A9).

### 2.5 Statistical Analyses

Age of first return migration could thus be assigned unambiguously for most species and allocated to one of the following four categories: (1) species that typically make a return migration for their first breeding period, and are therefore rarely caught or observed in Australia at this time; (2) species that typically spend their first breeding period in Australia and migrate north thereafter, in which the great majority (> 98%) of birds captured in Australia during the breeding period are one‐year old; (3) species that may also spend a second breeding period in Australia, in which small numbers of second‐year birds are also caught; and (4) species with long‐delayed maturity, in which most birds spend a second and at least some birds spend a third breeding period in Australia. However, seven out of 37 species could not be unambiguously assigned to a single category. To check that this did not bias our results, we also conducted analyses that excluded them (i.e., using a reduced dataset). We constructed two datasets: one in which each of these species was assigned the alternative age category least consistent with the trends identified from the reduced dataset, and one in which each of these species was assigned the alternative age category most consistent with the reduced dataset. Results of these sensitivity analyses are not presented here, but all were consistent in their assessments of the importance of predictor variables (Appendix A).

With the age of first return migration of each species being classified on an ordinal scale and having checked that the power transformation brought the distribution close to normality (Quinn and Keough 2020), we used the square root of this value as a continuous variable in subsequent modeling. Additionally, we carried out all analyses treating age of return migration as an ordered multinomial response. Doing so did not alter the conclusions (see Appendix A).

**FIGURE 1:**
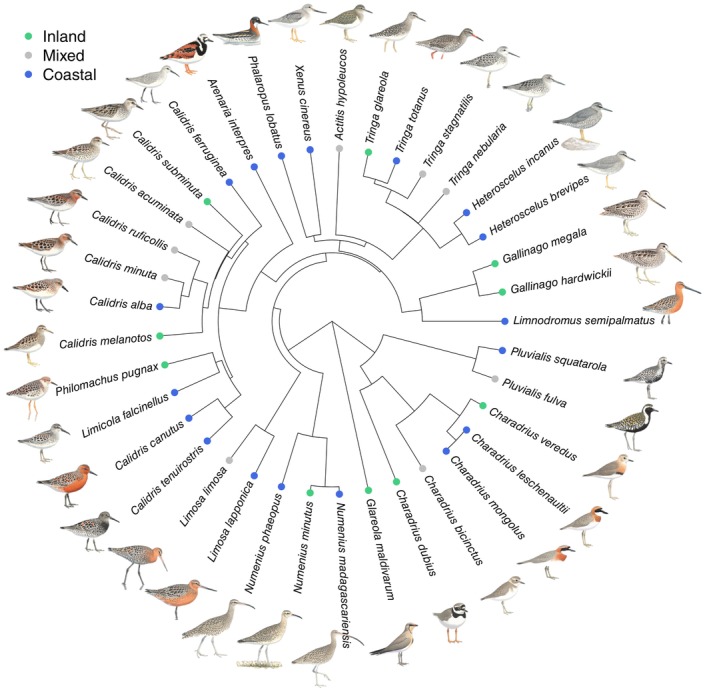
Maximum clade credibility tree for bird species included in the study. Colors of dots on terminal branches indicate different non‐breeding habitats used. Images reproduced from Menkhorst et al. (2017) with permission from CSIRO Publishing; they are for illustration only and are not to scale.

All statistical analyses were done in R version 4.2.1 (R Core Team 2022). Ensuring to take phylogeny into account when comparing traits across species (Felsenstein 1985; Harvey and Pagel 1991), we fitted Bayesian phylogenetic mixed models using the package ‘MCMCglmm’ (Hadfield 2010). Age of first return migration was included as the response variable (square root‐transformed and untransformed in order to fit models with Gaussian and ordinal family responses, respectively); non‐breeding habitat, migration distance, and breeding latitude as predictors and phylogeny as a random effect. We centered and scaled continuous predictors to unit variance to improve the interpretability of parameter estimates (Schielzeth 2010). We used the bird phylogeny from the ‘BirdTree project’ (Jetz et al. 2012; birdtree.org). To take into account phylogenetic uncertainty in our analysis, we built a maximum clade credibility (MCC) tree based on 10,000 phylogenetic trees from the Hackett backbone of the complete phylogeny of birds (Jetz et al. 2012). For all analyses, we used the MCC tree (Figure 1) and uninformative inverse Wishart priors for the residual and phylogenetic variance (Data S1). We ran two chains for 5,000,000 iterations with a thinning value of 2500 after a burn‐in of 100,000, resulting in effective sample sizes of 1960. We confirmed that the models had converged by examining parameter trace plots and posterior distributions. Potential‐scale reduction values were all < 1.1, and the autocorrelations of posterior samples were all < 0.1. All variables had variance inflation factors < 4. The effect of a given predictor was considered significant if zero was not included in the 95% CI. We estimated the importance of the species’ shared evolutionary history using heritability (*h* ^2^), a measure of phylogenetic signal ranging between 0 and 1, that can be calculated from the estimated phylogenetic variance in the model (Hadfield 2010). The interpretation of *h* ^2^ is identical to that of Pagel's *λ* in phylogenetic generalized least squares models (Freckleton et al. 2002), such that values close to 0 indicate that there is a negligible effect of phylogeny and values close to 1 that there is a strong phylogenetic signal in the data. We calculated the mode of *h* ^2^ across the entire posterior distribution. Finally, we calculated the deviance information criteria (DIC), a hierarchical generalization of the Akaike information criteria, for each Gaussian model and compared it to the paired ordinal models to compare the model ‘fit’ of each approach.

## 3 Results

Across all species, 865 known‐age retraps were captured in Australia during the breeding period when adults had migrated to other countries to breed (Table A3). The majority of the retraps were immatures (68.2% in their first year, 21.3% in their second or third year of life); only 10% were in their fourth year or older. Similar trends were apparent in the 13,600 shorebirds captured in Australia during the boreal breeding season and aged on moult and plumage attributes (Table A2). Ages of overseas recoveries of Australian‐banded birds, at least in some species, showed individual variation in the age of first return migration (Table A4). There was good support for a key assumption underlying our analyses of count and Atlas data: the number of migratory shorebirds occurring in Australia during the boreal breeding season is strongly correlated to the number of immatures present (Table A5). Over half of the species considered could be assigned unambiguously to a particular category of age of first return migration (Table A6).

**FIGURE 2:**
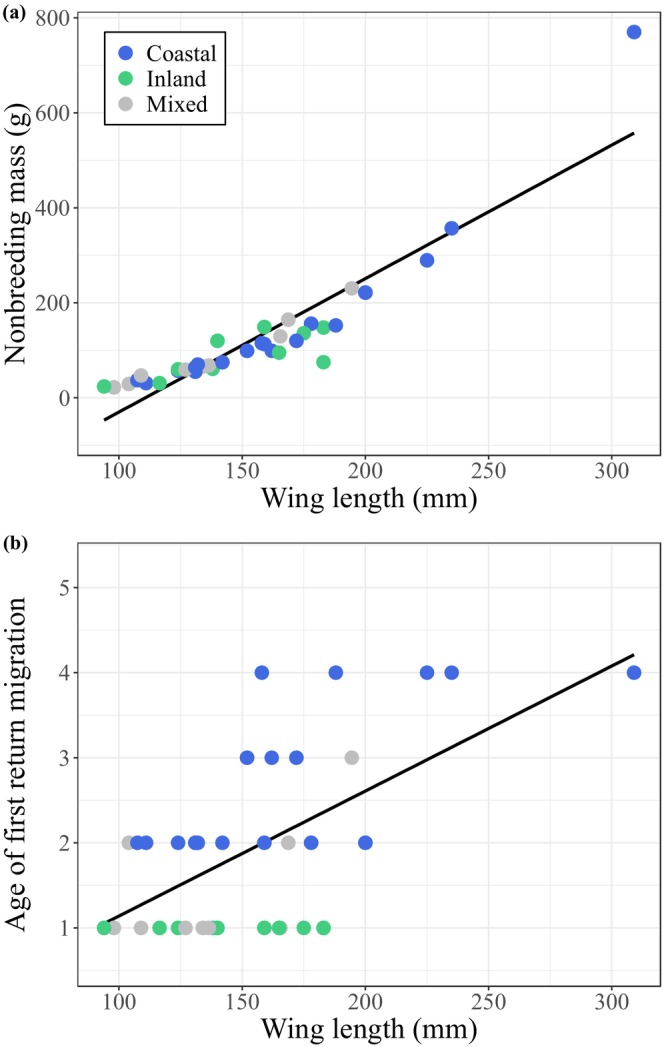
(a) Body mass plotted against wing length, and (b) age of first return migration plotted against wing length. To help visualize the trend in these data, simple linear models of the bivariate relationships are shown.

**FIGURE 3:**
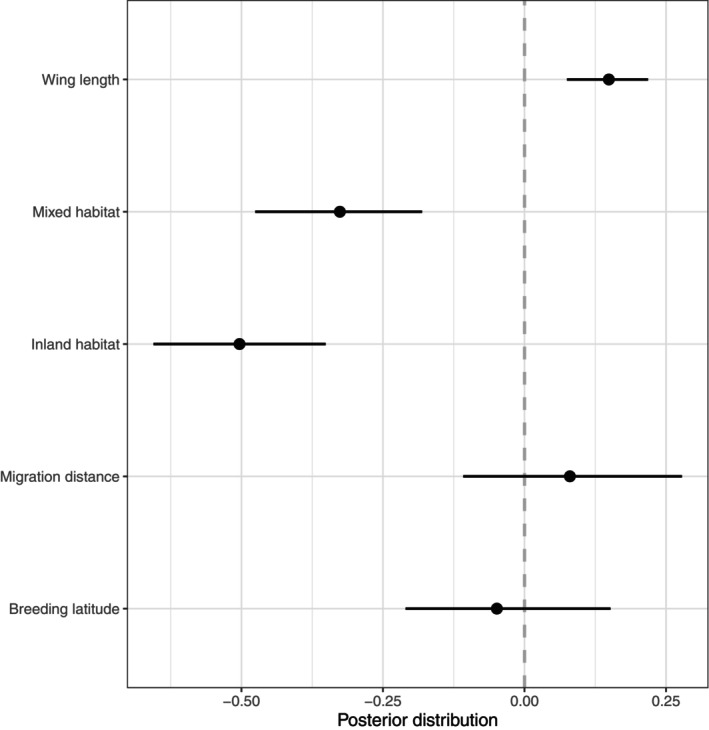
Effect of predictor variables on age of first return migration in 37 shorebird species. The posterior distribution of an independent variable with a negligible effect on age of first northward migration is expected to be centered on zero (dashed line); conversely, the distribution of an influential variable is expected to be substantially shifted from 0. Black dots indicate the posterior distribution, and horizontal lines indicate the 95% credible intervals. Reference category for habitat is ‘coastal’.

Of the 37 shorebird species of Australia, 11 belong to the inland habitat category, 9 to the mixed habitat category, and 17 to the coastal habitat category (Figure 1, Table 1). Strikingly, all 11 species of inland non‐breeding habitats, all individuals do their first northward migration as one‐year‐olds (age category 1), whereas the coastal species are distributed over the higher age categories 2–4 (Figure 2). Not surprisingly then, we find a strong statistical relationship between habitat used in the non‐breeding season and age of first return migration: coastal species delay maturity more than inland (Gaussian MCMCglmm: estimate = −0.503, CIs = −0.655 to −0.351) or ‘mixed’ species (estimate = −0.326, CIs = −0.476 to −0.181) (Figures 2 and 3). Moreover, we find a positive relationship between wing length (an indicator of body size) and age of first return migration (estimate = 0.149, CIs = −0.075 to −0.218) (Figures 2 and 3). The phylogenetic signal (measured as *h* ^2^) was substantial (*h* ^2^ posterior mode = 0.786, CIs = 0.222–0.967), suggesting that evolutionary history constrains delayed maturity. Age of first return migration was not correlated with migration distance or breeding latitude (all 95% CIs crossed zero; see Figure 3 and Appendix, Tables A7, A8, A9, A10, A11, A12, A13, A14, A15). Our model explained a significant proportion of the variance in age at first return migration, with a marginal *R* ^2^ = 0.64 and a conditional *R* ^2^ = 0.89 (DIC = −36.452). The results of the paired ordinal model were qualitatively identical, apart from an increase in the phylogenetic signal (*h* ^2^ posterior mode = 0.871, CIs = 0.575–0.962) and DIC value (DIC = 28.378). Again, the model had high explanatory power, explaining nearly all variation in age at first return migration (marginal *R* ^2^ = 0.99; conditional *R* ^2^ > 0.99).

**TABLE 1:** Life history attributes used in models of (relative) age of first return migration.

Species	Age of first return migration (ordinal variable)	Non‐breeding habitat	One‐way migration distance (km)	Breeding latitude (degrees)	Non‐breeding mass (g)	Wing length (mm)
Oriental pratincole *Glareola maldivorum*	1	Inland	6699	30	75	183
Little curlew *Numenius minutus*	1	Inland	9630	65	148	183
Eastern curlew *Numenius madagascariensis*	4	Coastal	10,216	55	770	309
Whimbrel *Numenius phaeopus*	4	Coastal	11,242	65	357	235
Black‐tailed godwit *Limosa limosa*	3	Mixed	9525	50	231	194.5
Bar‐tailed godwit *Limosa lapponica*	4	Coastal	11,724	70	290	225
Asian dowitcher *Limnodromus semipalmatus*	2 (3)	Coastal	8876	54	156	178
Latham's snipe *Gallinago hardwickii*	1	Inland	10,049	42	149	159
Swinhoe's Snipe *Gallinago megala*	1	Inland	9211	58.5	120	140
Little stint *Calidris minuta*	1	Mixed	11,619	70	22	98
Red‐necked stint *Calidris ruficollis*	2	Mixed	11,514	65	29	104
Long‐toed stint *Calidris subminuta*	1	Inland	10,195	55	24	94
Pectoral sandpiper *Calidris melanotus*	1	Inland	11,514	70	61	138
Sanderling *Calidris alba*	2 (1)	Coastal	11,933	70	57	124
Sharp‐tailed sandpiper *Calidris acuminata*	1	Mixed	11,096	65	66	134
Broad‐billed sandpiper *Limicola falcinellus*	2	Coastal	11,305	68	37	107.5
Ruff *Philomachus pugnax*	1	Inland	10,258	55	136	175
Great knot *Calidris tenuirostris*	4	Coastal	11,305	63	153	188
Red knot *Calidris canutus*	4	Coastal	11,514	70	115	158
Curlew sandpiper *Calidris ferruginea*	2	Coastal	11,786	70	55	131
Ruddy turnstone *Arenaria interpres*	3	Coastal	11,514	70	99	152
Marsh sandpiper *Tringa stagnatilis*	1 (2)	Mixed	9839	53	68	136.5
Common redshank *Tringa totanus*	2	Coastal	7955	40	113	159
Common greenshank *Tringa nebularia*	2	Mixed	10,530	60	165	168.7
Gray‐tailed tattler *Tringa brevipes*	3	Coastal	10,782	60	99	162
Wandering tattler *Tringa incanus*	3 (4)	Coastal	12,247	65	120	172
Wood sandpiper *Tringa glareola*	1	Inland	10,195	55	60	124
Red‐necked phalarope *Phalaropus lobatus*	2 (1)	Coastal	10,782	60	31	111
Common sandpiper *Actitis hypoleucos*	1	Mixed	10,049	55	47	109
Terek sandpiper *Xenus cinereus*	2	Coastal	11,221	65	70	132
Gray plover *Pluvialis squatarola*	2	Coastal	11,828	74	222	200
Pacific golden plover *Pluvialis fulva*	1 (2)	Mixed	12,456	72	129	165.5
Greater sand plover *Charadrius leschenaultii*	2	Coastal	9190	45	75	142
Little ringed plover *Charadrius dubius*	1	Inland	8165	47	31	116.5
Double‐banded plover *Charadrius bicinctus*	1	Mixed	2219	−43	59	127
Lesser sand plover *Charadrius mongolus*	2 (3)	Coastal	10,153	55	64	131
Oriental plover *Charadrius veredus*	1	Inland	7662	43	95	165

*Note:* The age categories assigned are: (1) Species that typically migrate north for their first breeding period, when ~12 months old; (2) Species that spend their first breeding period in Australia, first migrate north when 2 years (~24 months) old, and migrate annually thereafter; (3) Species that may also spend a second breeding period in Australia; and (4) Species with long‐delayed maturity, in which most birds spend a second and at least some birds spend a third breeding period in Australia. Age between parentheses indicates the alternative age category consistent with the reduced dataset. Non‐breeding habitat is assigned as inland, coastal, or ‘mixed’ for species that occur regularly in both habitats.

Results are qualitatively similar to those presented here (identifying non‐breeding habitat as the strongest correlate with age of first return migration) when: (1) body mass, instead of wing chord, is used as a measure of size, (2) the reduced dataset is used, or (3) alternative age categories least and most consistent with the reduced dataset are used (see Appendix, Tables A7, A8, A9, A10, A11, A12, A13, A14, A15).

## 4 Discussion

Our study indicates that the age of maturity in migratory shorebirds is correlated with environmental attributes, with clear differences between migratory species that spend the non‐breeding season in coastal rather than in inland wetland habitats. It demonstrates that the coastal‐inland dichotomy first identified by Piersma (1997) and elaborated in Piersma (2003) not only associates with physical and ecological attributes, but also with one of four fundamental life‐table variables (Stahl and Oli 2006).

Shorebirds with delayed maturity are missing breeding opportunities, and if their populations are to remain stable, it is inevitable that there will be concomitant differences in other life‐table variables. It is unlikely that shorebirds could compensate for lost breeding opportunities in early life through increased fecundity, as the fecundity of shorebirds is capped by the maximum clutch size of four (Arnold 1999). We therefore expect delayed maturity in shorebirds to be associated with increased pre‐reproductive survival, with increased adult survival, or both. There is evidence in several shorebird species that pre‐reproductive survival of shorebirds that remain on the non‐breeding grounds is higher than that of shorebirds that carry out a return migration (Piersma et al. 2016; Tavera et al. 2020; Ydenberg 2025). In addition, we predict that once enough measurements of robust annual adult survival rates have been obtained from non‐declining populations of migratory shorebirds, they will prove to be positively correlated with age at first breeding attempt. We note that in Méndez et al.'s (2018) review of shorebird annual survival rates (which did not assess non‐breeding habitat as a potential correlate), three genera stood out for their high survival rates: *Haematopus* (Oystercatchers), a genus renowned for its delayed maturity (Ens et al. 1995) and *Limosa* (godwits) and *Numenius* (curlews and whimbrels), the genera with the longest‐delayed maturity in our study.

Delayed maturity is characteristic of some bird species, with the first breeding attempt being delayed for some time (often several years) beyond the age when they are fully grown and apparently physiologically capable of breeding (Newton 1998; Cam et al. 2005). Proposed explanations fall broadly into two categories (Williams 1966; Curio 1983). According to *constraint* hypotheses, individuals that delay maturity are unable to start breeding at an earlier age because of a shortage of some resource. Under *restraint* hypotheses, breeding reduces survival, particularly when young, to the extent that birds that refrain from breeding when young will live longer and have greater lifetime reproductive success. Ydenberg's (2025) refinement of the restraint hypothesis points out that the success of the first breeding attempt of migratory shorebirds is probably only half that of adults, so the trade‐offs between survival and breeding differ between adult and younger birds. Ydenberg (2025) suggests that migration, or deferring return migration for a year, may be facultative alternatives for young shorebirds; for example, the decision of whether or not to set off on return migration could be based on the body condition achieved by a particular date. We consider this a plausible scenario in second‐ or third‐year shorebirds, in which the timing of flight‐feather and pre‐breeding moults is like those of adults.

In contrast, some shorebirds may have evolved *constraints* that greatly reduce their chance of successfully completing a return migration or breeding in their first year. In red‐necked stints, for example, adults undergo substantial pre‐departure mass gains from March to May but first‐year individuals do not show any detectable mass gains at that time of year (Rogers et al. 1996); first‐year immatures replace no flight feathers at the time of year (~Oct–Jan) when adults carry out complete flight‐feather moult. At the time of year (Feb–May) when adult red‐necked stints moult into brightly colored plumage, first‐year birds only carry out limited pre‐alternate body moult, and the resultant plumage is drab rather than brightly colored (Higgins and Davies 1996). As a result, by late April when adult red‐necked stints have left southern Australia on return migration (Lisovski et al. 2021), first‐year birds have worn flight feathers, do not have the fuel stores required to migrate thousands of kilometers, and they do not have the body plumage necessary for breeding success. Several other Australian shorebird species also have moult schedules in the first year that would likely be maladaptive to individuals that attempted a return migration in the first year (Higgins and Davies 1996). The extent to which moult cycles constrain variation in the age of first return migration and breeding merits further study.

Several environmental factors have been proposed to explain the variation in the age of maturity. McNeil et al. (1994) suggested that high rates of trematode infestation in subadult shorebirds were responsible; Wille and Klaassen (2022) also noted that juvenile shorebirds are likely to be more susceptible to parasite or viral infection than adults. However, it is difficult to envisage how such infections would delay maturity more in coastal than in inland species, given that: (1) marine‐ or freshwater‐restricted shorebirds have similar helminth species richness, this being highest in species with a mixed wintering habitat use (Gutiérrez et al. 2017); (2) the intensity, prevalence, or diversity of blood parasites are typically lower in intertidal habitats than in freshwater ones (Piersma 1997; Figuerola 1999; Mendes et al. 2005; Yohannes et al. 2009; Clark et al. 2016; Soares et al. 2016); and (3) in an Argentinian study, there was no evidence of a weakened immune system, high loads of blood parasites, or high stress levels that could explain the poorer migratory condition of adults that remain in the non‐breeding grounds and miss a breeding opportunity (Martínez‐Curci et al. 2020).

Consistent with the positive relationships between wing length, age of first return migration, and longevity previously reported by Summers et al. (1995), here also we find that body size is correlated with delayed maturity. This is no surprise, as body size is a good predictor of annual survival of shorebirds (Méndez et al. 2018), which complements abundant evidence that in all groups of birds and mammals, the larger species live longer than the smaller ones (Lindstedt and Calder 1981; Newton 1998; Saether 1989; Speakman 2005; Healy et al. 2014; Soriano‐Redondo et al. 2020), with strong negative relationships between age of maturity and annual adult mortality (Ricklefs 2000; Bennett and Owens 2002).

Delayed maturity could represent a strategy to maximize reproductive success in circumstances where “the survival advantage compensates in fitness terms for the reproduction foregone by doing so” (Ydenberg 2025). In semipalmated sandpipers *Calidris pusilla* , an increasing danger of depredation by falcons at stopover sites (where falcons have increased in numbers) is argued to have increased the likelihood of deferring return migration and the resultant loss of a breeding season (Ydenberg and Hemerik 2024). More generally, young adults are in competition with older adults for limited resources, particularly for food and breeding opportunities (Hawkins et al. 2012). Young animals are typically less efficient at foraging than adults (Wunderle Jr. 1991), but such age differences have been found in shorebirds foraging in both coastal (Groves 1978; Puttick 1978, 1979; Goss‐Custard and Durell 1987; Turpie and Hockey 1993) and non‐coastal habitats (Burger 1980; Espin et al. 1983; Hockey et al. 1998). Hockey et al. (1998) proposed that the lower foraging proficiencies of immatures on the non‐breeding grounds delay their pre‐migratory mass gain, so they cannot attain optimal departure mass on the tight schedules required to breed successfully during the brief boreal summer. If there was virtually no chance of breeding success on the first attempt, then there would be selection for delayed maturity if it resulted in only a slightly higher probability of survival in the first years of life. Building on this theory, Hockey et al. (1998) further suggested that maturity is likely to be delayed longer in shorebird species that migrate further and therefore need more fuel. In our study, we did not find a relationship between migration distance and age of first return migration. This echoes the absence of a negative correlation between migration distance and adult annual survival (Conklin et al. 2017). However, we note that both analyses were restricted to populations that migrate very long distances to Australasia; the age of return migration in shorebirds of the East Asian Australasian shorebirds that spend their non‐breeding season in north‐temperate latitudes has yet to be investigated. In other flyways, there is evidence for several species that immature shorebirds that migrate into the southern hemisphere delay their return migration longer than shorebirds that only migrate as far south as north‐temperate regions (Summers et al. 1995; Fernandez et al. 2004; O'Hara et al. 2005; Tavera et al. 2020).

We think that the strong effect of non‐breeding habitat on age of first return migration fits in well with the theory that lower foraging proficiency of immatures is a cause of delayed maturity. It implies that shorebirds may take longer to learn how to forage efficiently in intertidal feeding areas than in inland freshwater habitats. This does not necessarily mean that foraging in inland wetlands is without challenges. Most inland habitats in Australia used by large numbers of shorebirds are unpredictable temporary wetlands (Hansen et al. 2016); habitat availability inland varies substantially from year to year (Clemens et al. 2021) and accessing temporary Australian wetlands in peak condition often requires movements of hundreds of kilometers during the non‐breeding season (Pedler et al. 2014). However, we think there are some challenges particular to foraging in the intertidal zone. First, there are time constraints; intertidal flats are regularly immersed by high tides, and for some 12 h per day, tidal flats below mean sea level cannot be used as feeding sites by shorebirds. This would limit the capacity of immature coastal shorebirds (with inferior foraging skills) to increase their daily food intake by extending their foraging hours. Moreover, there is some evidence that young animals take longer to develop adult levels of foraging proficiency if their prey is difficult to find or handle (Wunderle Jr. 1991). We think it is plausible that it takes shorebirds a relatively long time to learn how to exploit the food hidden in tidal flats; while inland shorebirds largely forage on dipterid larvae and Coleoptera, shorebirds of tidal flats have diets often dominated by species that burrow more deeply into the substrate, such as bivalves, amphipods, and polychaetes (Skagen and Oman 1996). We note that the species with the longest‐delayed maturity in our study all target large intertidal prey capable of burrowing out of reach of shorebirds: e.g., the sentinel crabs and mantis shrimps hunted by eastern curlew and whimbrel (Dann 1993, 2014), the bivalves hunted by great and red knots (Tulp and de Goeij 1994), and the large polychaetes hunted by bar‐tailed godwits (Duijns et al. 2013).

We found that phylogenetic signal estimates were considerable across models (*h* ^2^ posterior mode was > 0.7 in both Gaussian and ordinal models). This is consistent with the notion that shorebirds' life‐history traits such as incubation (Bulla et al. 2016) and moult rhythms (Dietz et al. 2015) are largely conserved among related species. Because maturity is a life‐history trait expected to correlate with evolution, it is necessary to incorporate correlational structures reflecting phylogenetic relationships in regression analyses (Pagel 1994). Nevertheless, phylogenetic effects did not mask the ecological patterns identified here. Future studies using individual‐level trait data will be necessary to examine how accounting for intraspecific trait variation influences the detection of these patterns as well as to infer the processes underlying delayed maturity.

## Author Contributions

**Danny I. Rogers:** conceptualization (equal), formal analysis (equal), investigation (equal), methodology (equal), writing – original draft (lead), writing – review and editing (equal). **Theunis Piersma:** conceptualization (equal), writing – original draft (equal), writing – review and editing (lead). **Clive D. T. Minton:** investigation (equal), methodology (equal). **Adrian N. Boyle:** investigation (equal), methodology (equal), writing – review and editing (supporting). **Chris J. Hassell:** data curation (equal), investigation (equal), methodology (equal), writing – review and editing (supporting). **Ken G. Rogers:** conceptualization (equal), formal analysis (equal), methodology (equal). **Andrew Silcocks:** investigation (equal), resources (supporting). **Jorge S. Gutiérrez:** formal analysis (lead), methodology (equal), writing – review and editing (equal).

## Conflicts of Interest

The authors declare no conflicts of interest.

## Supporting information


Data S1

## Data Availability

All basic data are provided in the Appendix A, whereas all the scripts for the comparative analysis have been uploaded as Supporting Information Data S1.

## Appendix A

The aging based on plumage and moult characters indicated that the great majority of shorebirds captured in both Victoria and north‐western Australia during the breeding period (Table A1) were immatures (Table A2). This was also true of known‐age retraps (Table A2). When comparing the ages of known‐age retraps with the ages they were assigned to on the basis of moult and plumage characters when retrapped, most aging errors involved confusion of adults and birds in the second or third breeding period (Table A3). Errors involving incorrect aging of birds in their first breeding period were made in only two bar‐tailed godwits and eight great knots in north‐western Australia, and in three red knots, one curlew sandpiper, and two red‐necked stints in south‐eastern Australia. In general, moult‐based aging of birds in their first breeding period thus appeared to be reasonably accurate. For some species, different age ratios were found in morphologically aged samples and known‐age retraps, but we attribute this to annual variation in the number of first‐year birds that had been banded and were thus available for recapture.

**TABLE A1:** Cut‐off dates of breeding periods used in this study. These adjusted definitions allowed us to increase sample sizes for some comparisons, but in all cases, we chose conservative cut‐off dates that we could use for both north‐western and south‐eastern Australia without including typical breeding adults in the samples from ‘breeding periods’.

Cut‐off dates	Applied to the following species
1 May–15 July	Eastern curlew; greater sand plover
15 May–15 July	Long‐toed stint; marsh, wood, and common sandpipers.
25 May–9 Aug.	Red knot (see Battley et al. 2005 for details of late departures)
15 May–1 Aug.	Little ringed plover; oriental plover; oriental pratincole
15 Sep–1 Feb.	Double‐banded plover
15 May–9 Aug.	All other species

**TABLE A2:** Age distribution of shorebirds during the ‘breeding period’ in north‐western Australia and south‐eastern Australia, based on morphological age characteristics in catches made from 1980–2005. The percentages of birds aged as being in their first, second, or third breeding periods (BP) are given, as are the percentages of birds aged as 2+ or 3+ (i.e., in their second year or older, and apparently in adult plumage). The final column gives the probability that the proportion of birds in their first breeding period was the same in north‐western and south‐eastern Australia.

Species	North‐Western Australia						South‐Eastern Australia						NWA vs. Vic. (*p*)
	Total banded	*N* during BP	1st BP (%)	2nd BP (%)	3rd BP (%)	Apparent adult (%)	Total banded	Breeding period sample	1st BP (%)	2nd BP (%)	3rd BP (%)	Apparent adult (%)	
Eastern Curlew	155	17	88.2	0.0	0.0	11.8	856	59	45.8	18.6	3.4	32.2	0.101
Whimbrel	278	32	62.5	34.4	0.0	3.1	24	1	100.0	0.0	0.0	0.0	0.316
Black‐tailed Godwit	701	99	85.9	5.1	0.0	9.1							
Bar‐tailed Godwit	11,314	970	60.8	29.4	4.5	5.3	3271	534	74.9	13.9	8.2	3.0	< 0.0001
Asian Dowitcher	107	37	97.3	0.0	0.0	2.7							
Red‐necked Stint	14,584	623	98.0	0.0	0.0	2.0	113,378	4036	98.7	0.0	0.0	1.3	0.999
Sanderling	659	9	100.0	0.0	0.0	0.0							
Sharp‐tailed Sandpiper	1012	0					6886	2	100.0	0.0	0.0	0.0	
Broad‐billed Sandpiper	1331	20	100.0	0.0	0.0	0.0							
Red Knot	5525	771	72.0	10.5	1.7	15.8	4355	1016	90.6	7.5	0.0	2.0	< 0.0001
Great Knot	18,727	2023	66.0	21.7	1.7	10.6	638	62	61.3	37.1	0.0	1.6	0.074
Curlew Sandpiper	10,014	1043	98.3	0.1	0.0	1.6%	28,128	1158	99.7	0.0	0.0	0.3	0.999
Ruddy Turnstone	1777	99	91.9	5.1	0.0	3.0	3050	52	100.0	0.0	0.0	0.0	0.998
Common Greenshank	184	13	100.0	0.0	0.0	0.0							
Gray‐tailed Tattler	6371	450	98.2	0.7	0.0	1.1	40	4	100.0	0.0	0.0	0.0	0.999
Terek Sandpiper	6470	209	99.0	0.0	0.0	1.0							
Gray Plover	302	1	100.0	0.0	0.0	0.0	161	2	100.0	0.0	0.0	0.0	0.999
Greater Sand Plover	10,804	252	98.8	0.8	0.0	0.4	34	1	100.0	0.0	0.0	0.0	0.999
Double‐banded Plover	0	0					4397	3	100.0	0.0	0.0	0.0	
Lesser Sand Plover	527	7	100.0	0.0	0.0	0.0	150	7	57.1	0.0	0.0	42.9	0.794

**TABLE A3:** Age distribution of north‐western and south‐eastern Australian shorebirds during the ‘breeding period’, based on known‐age retraps within Australia. The percentages of birds in their first, second or third breeding periods are given, as are the number of aging errors – i.e., cases in which age assigned on plumage or moult characters was inconsistent with the correct age based on band history. Retraps aged as ‘adult’ were at least 4 years old.

	North‐Western Australia						South‐Eastern Australia					
	*N* retraps	Aging errors	1st (%)	2nd (%)	3rd (%)	Adult (%)	*N* retraps	Aging errors	1st (%)	2nd (%)	3rd (%)	Adult (%)
Eastern Curlew	0						1	0	0.0	0.0	0.0	100.0
Bar‐tailed Godwit	73	9	34.2	37.0	8.2	20.5	19	0	52.6	26.3	5.3	15.8
Asian Dowitcher	1	0	100.0	0.0	0.0	0.0	0					
Red‐necked Stint	12	0	91.7	0.0	0.0	8.3	197	2	95.4	2.5	0.5	1.5
Great Knot	103	20	58.3	29.1	3.9	8.7	1	0	100.0	0.0	0.0	0.0
Red Knot	22	5	63.6	4.5	9.1	22.7	44	3	79.5	11.4	2.3	6.8
Curlew Sandpiper	39	0	94.9	0.0	0.0	5.1	54	1	100.0	0.0	0.0	0.0
Ruddy Turnstone	0						20	0	100.0	0.0	0.0	0.0
Gray‐tailed Tattler	12	1	91.7	8.3	0.0	0.0	0					
Terek Sandpiper	5	0	100.0	0.0	0.0	0.0	0					
Greater Sand Plover	15	0	93.3	0.0	0.0	6.7	0					
Double‐banded Plover	0						1	0	100.0	0.0	0.0	0.0
Lesser Sand Plover	3	0	100.0	0.0	0.0	0.0	2	0	0.0	50.0	50.0	0.0

Ages of overseas recoveries of Australian‐banded birds (Table A4) showed that in some species, there was individual variation in the age of first return migration. Retraps demonstrated that some second‐year great knots and red knots, and some third‐year bar‐tailed godwits remain within Australia; however, some second‐year red and great knots and some third‐year bar‐tailed godwits were also recovered on migration. In species in which the great majority of birds found in the breeding period within Australia were in their first year, there were generally no first‐year overseas recoveries. Exceptions were found in double‐banded plovers (the only species in Table A4 that typically breeds in its first year; recoveries were from the breeding grounds). A sanderling recovered in Japan on 1 August 2005 was aged as a first‐year bird when initially captured in South Australia on 15 March 2005. Finally, a first‐year Terek sandpiper recovered in the Philippines in August 2000 had been banded in north‐western Australia on 19 May 2000. As this bird was initially captured after the usual departure date of adults on return migration, it is likely that its movement to the Philippines was related to subadult dispersal rather than a genuine attempt at return migration. To estimate the age of first return migration in species that had not been banded in large numbers, we examined the ratio of birds in the breeding and non‐breeding periods in the Atlas and AWSG Population Monitoring Project datasets (Table A5).

**TABLE A4:** Numbers of known‐age recoveries from breeding areas or staging sites outside Australia.

Species	*N* of known‐age recoveries	Years after hatching			
		1	2	3	4+
Banded in north‐western Australia					
Whimbrel	1		1		0
Bar‐tailed Godwit	19			2	17
Red‐necked Stint	2			1	1
Great Knot	84		8	17	59
Red Knot	3		2		1
Curlew Sandpiper	1				1
Ruddy Turnstone	1				1
Gray‐tailed Tattler	5		2		3
Terek Sandpiper	4	1		1	2
Greater Sand Plover	2			2	0
Banded in south‐eastern Australia					
Red‐necked Stint	18		5	4	9
Sanderling	1	1			0
Great Knot	4		2		2
Red Knot	8		1	2	5
Curlew Sandpiper	4				4
Ruddy Turnstone	3		1	1	1
Double‐banded Plover	11	2	3	1	5

**TABLE A5:** Proportions of shorebird abundance in breeding periods relative to non‐breeding periods, as determined by Atlas records and AWSG Population Monitoring Project (PMP) counts. We divided these proportions into four separate groups using *K*‐means clustering.

Species	Total Atlas records	Atlas ratio (%)	Total on PMP counts	PMP ratio (%)
Oriental Pratincole	120	1.0	940	0.0
Little Curlew	237	3.2	1344	3.5
Eastern Curlew	4655	75.6	131,005	23.4
Whimbrel	4073	82.7	29,239	45.4
Black‐tailed Godwit	661	67.4	31,534	20.6
Bar‐tailed Godwit	4711	93.2	926,307	18.4
Asian Dowitcher	40	50.8	96	190.9
Latham's Snipe	2063	4.6	3204	0.0
Pin‐tailed Snipe	2	0.0	2	0.0
Swinhoe's Snipe	16	0.0	5	0.0
Little Stint	4	0.0	3	0.0
Red‐necked Stint	4663	73.8	1,332,843	10.2
Long‐toed Stint	100	0.0	98	2.1
Pectoral Sandpiper	131	0.0	283	0.0
Sanderling	574	40.2	19,628	4.2
Sharp‐tailed Sandpiper	3433	6.1	353,930	0.2
Broad‐billed Sandpiper	82	30.6	0	
Ruff	46	0.0	40	0.0
Red Knot	814	48.1	190,019	34.6
Great Knot	1238	66.4	519,479	14.8
Curlew Sandpiper	2422	45.7	520,497	7.6
Ruddy Turnstone	2096	66.3	62,739	14.2
Marsh Sandpiper	1755	4.5	8813	5.0
Common Redshank	14	27.1	2	100.0
Common Greenshank	4558	39.1	50,018	13.0
Gray‐tailed Tattler	2339	79.2	59,708	28.9
Wandering Tattler	127	73.0	7	16.7
Wood Sandpiper	529	0.1	266	1.5
Red‐necked Phalarope	34	43.9	5	0.0
Common Sandpiper	1948	6.4	751	3.9
Terek Sandpiper	693	51.7	29,026	16.9
Gray Plover	935	29.7	34,964	9.5
Pacific Golden Plover	2188	25.6	30,836	2.8
Greater Sand Plover	907	24.5	88,411	18.0
Little Ringed Plover	17	0.0	2	0.0
Double‐banded Plover	1160	6.7	64,967	1.7
Lesser Sand Plover	913	56.7	46,377	12.4
Oriental Plover	109	0.6	41,705	0.0

Synthesizing the information from the different data sets, in Table A6 we bring the allocations of the species to the four age of maturity categories together. Over half of the species considered could be assigned unambiguously to a particular category of age of first return migration, as they fitted into the same cluster regardless of the dataset used. This was the case for most species in which the first return migration was not delayed. In species in which banding data and winter‐summer ratios generated slightly conflicting results, we regarded the banding data as superior as it was a direct measure of age structure during the breeding period. In red‐necked stints, for example, Atlas data revealed a high ratio of austral winter to austral summer reports, although there were abundant banding data to confirm that this species only spends its first breeding period in Australia. We believe the anomalous result from Atlas data to have been caused by the exceptionally high breeding success that red‐necked stints experienced in the period (1998–2002) when the Atlas data were collected (Minton et al. 2005). First‐year birds were abundant as a result, and austral winter counts were correspondingly high.

**TABLE A6:** The category of age of first return migration was identified from the Atlas database and PMP counts (identified by using *K*‐means clustering to break the columns of ratios in Table A5 into 4 clusters), and the age category indicated by moult‐based aging or known‐age retraps of birds caught in Australia during the breeding periods. The age of first return migration used in the statistical analyses is given in the final column.

Species	Method				Category age of first return migration assigned to
	Atlas	PMP	Age	Band	
Oriental Pratincole	1	1	1		1
Little Curlew	1	1	1		1
Eastern Curlew	4	3	4	3	4
Whimbrel	4	4	4	3	4
Black‐tailed Godwit	3	3	3		3
Bar‐tailed Godwit	4	3	4	4	4
Asian Dowitcher	3		2	2	2 or 3
Latham's Snipe	1	1	1		1
Pin‐tailed Snipe	1	1			n.a.
Swinhoe's Snipe	1	1			1
Little Stint	1	1			1
Red‐necked Stint	4	2	2	2	2
Long‐toed Stint	1	1	1		1
Pectoral Sandpiper	1	1			1
Sanderling	2	1	2		1 or 2
Sharp‐tailed Sandpiper	1	1	1		1
Broad‐billed Sandpiper	2		2		2
Ruff	1	1			1
Red Knot	3	4	4	4	4
Great Knot	3	2	4	4	4
Curlew Sandpiper	2	2	2	2	2
Ruddy Turnstone	3	2	3	2	3
Marsh Sandpiper	1	1	1		1 or 2
Common Redshank	2				2
Common Greenshank	2	2	2		2
Gray‐tailed Tattler	4	4	2	3	3
Wandering Tattler	4	3			3 or 4
Wood Sandpiper	1	1	1		1
Red‐necked Phalarope	2	1			2 or 1
Common Sandpiper	1	1			1
Terek Sandpiper	3	3	2	2	2
Gray Plover	2	2	2		2
Pacific Golden Plover	2	1	1		1 or 2
Greater Sand Plover	2	3	2	2	2
Little Ringed Plover	1	1			1
Double‐banded Plover	1	1	1		1
Lesser Sand Plover	3	2	2	3	2 or 3
Oriental Plover	1	1	1		1

In some species, we could not assign the age of first return migration with confidence. There were too few records of pin‐tailed snipe to form any conclusion, and this species was excluded from further analysis. Samples were also small for little stint, but this species was treated as carrying out its first return migration when 1 year‐old, because all of the 60 Australian records of this species were recorded in Higgins and Davies (1996) were made during the non‐breeding period; no records were made in Australia during the breeding period, although this is the time of year that little stints are most easily identified (in the non‐breeding period it is easily overlooked because the non‐breeding plumages of red‐necked and little stints are very similar; e.g., Higgins and Davies 1996).

In seven species, arguments could be put forward for assignation to either of two categories. The low numbers of Sanderling and Marsh Sandpiper seen in the breeding period suggest the majority migrate north when one‐year old, yet the limited pre‐migratory mass gain occurring in some first‐year individuals in March–May and their limited acquisition of breeding plumage (Gosbell and Minton 2001; AWSG, unpubl. data) appear inconsistent with all migrating to the breeding grounds. Pacific golden plovers undergo extensive pre‐migratory mass gain at the end of their first austral summer (Barter 1988) and numbers in Australia during the breeding period have been considered too low to suggest delayed maturity (Marchant and Higgins 1993); yet there was a relatively high Atlas reporting rate during the breeding period. Records of red‐necked phalarope in the Atlas dataset suggested this species spends its first breeding period in the non‐breeding grounds, but observations of first‐year birds on the breeding grounds (Schamel and Tracy 1988) are inconsistent with this interpretation (Higgins and Davies 1996). Asian dowitchers captured during the breeding period were all in their first austral winter, but samples were quite small, and the high numbers of this species in the breeding period relative to those in the non‐breeding period may imply long‐delayed maturity (however, count data for this species come largely from Roebuck Bay, a site where Asian dowitchers are difficult to survey, Rogers et al. 2000). In lesser sand plovers, known‐age retraps of second‐ and third‐year birds in Victoria may have been misleading, as the only two records were of the same individual; it may have been ill or otherwise aberrant.

In addition to the main analyses presented in the main text (see Tables A7 and A8 below), we also ran sensitivity analyses using (1) body mass instead of wind length (Table A9); (2) alternative age categories consistent with a reduced dataset where species were unambiguously assigned to a single category (Tables A10, A11); and (3) the reduced dataset (Tables A12, A13, A14, A15).

**TABLE A7:** Results from the Gaussian MCMCglmm model are presented in the main text. Significant predictors (i.e., their 95% CIs did not overlap with zero) are in bold.

Parameter	Estimate	Lower CI	Upper CI	pMCMC
Intercept	1.531	1.321	1.727	0.001
**Inland habitat**	**−0.503**	**−0.655**	**−0.351**	**0.001**
**Mixed habitat**	**−0.326**	**−0.476**	**−0.181**	**0.001**
Breeding latitude	−0.049	−0.210	0.152	0.585
Migration distance	0.080	−0.108	0.278	0.388
**Wing length**	**0.148**	**0.075**	**0.218**	**0.001**

*Note:* Reference category for habitat is ‘coastal’.

**TABLE A8:** Results from the ordinal MCMCglmm model are presented in the main text. Significant predictors (i.e., their 95% CIs did not overlap with zero) are in bold.

Parameter	Estimate	Lower CI	Upper CI	pMCMC
Intercept	5.756	1.488	10.945	0.003
**Inland habitat**	**−322.531**	**−692.500**	**−8.353**	**< 0.001**
**Mixed habitat**	**−5.669**	**−10.117**	**−1.895**	**< 0.001**
Breeding latitude	−1.632	−7.252	4.520	0.537
Migration distance	2.748	−2.297	8.370	0.271
**Wing length**	**4.520448**	**1.852**	**7.868**	**< 0.001**

*Note:* Reference category for habitat is ‘coastal’.

**TABLE A9:** Results from the Gaussian MCMCglmm model using non‐breeding body mass instead of wind length. Significant predictors (i.e., their 95% CIs did not overlap with zero) are in bold.

Parameter	Estimate	Lower CI	Upper CI	pMCMC
Intercept	1.559	1.343	1.786	0.001
**Inland habitat**	**−0.497**	**−0.665**	**−0.329**	**< 0.001**
**Mixed habitat**	**−0.353**	**−0.509**	**−0.199**	**< 0.001**
Breeding latitude	−0.023	−0.215	0.159	0.832
Migration distance	0.058	−0.131	0.264	0.582
**Body mass**	**0.123**	**0.054**	**0.200**	**< 0.001**

*Note:* Reference category for habitat is ‘coastal’.

**TABLE A10:** Results from the Gaussian MCMCglmm model using the alternative age. Significant predictors (i.e., their 95% CIs did not overlap with zero) are in bold.

Parameter	Estimate	Lower CI	Upper CI	pMCMC
Intercept	1.542	1.314	1.737	0.001
**Inland habitat**	**−0.482**	**−0.691**	**−0.286**	**< 0.001**
Mixed habitat	0.191	−0.407	0.003	0.066
Breeding latitude	−0.035	−0.281	0.180	0.760
Migration distance	0.103	−0.130	0.340	0.409
**Wing length**	**0.179**	**0.079**	**0.260**	**< 0.001**

*Note:* Reference category for habitat is ‘coastal’.

**TABLE A11:** Results from the ordinal MCMCglmm model using the alternative age. Significant predictors (i.e., their 95% CIs did not overlap with zero) are in bold.

Parameter	Estimate	Lower CI	Upper CI	pMCMC
Intercept	3.037	0.833	5.827	0.010
**Inland habitat**	**−96.843**	**−206.217**	**−5.106**	**< 0.001**
Mixed habitat	−1.762	−3.591	0.103	0.061
Breeding latitude	−0.992	−5.398	3.100	0.636
Migration distance	1.989	−1.643	6.149	0.347
**Wing length**	**3.081**	**1.293**	**5.058**	**< 0.001**

*Note:* Reference category for habitat is ‘coastal’.

**TABLE A12:** Results from the Gaussian MCMCglmm model using the reduced dataset. Significant predictors (i.e., their 95% CIs did not overlap with zero) are in bold.

Parameter	Estimate	Lower CI	Upper CI	pMCMC
Intercept	1.570	1.376	1.767	0.001
**Inland habitat**	**−0.526**	**−0.710**	**−0.321**	**< 0.001**
**Mixed habitat**	**−0.297**	**−0.509**	**−0.102**	**0.005**
Breeding latitude	−0.038	−0.273	0.223	0.770
Migration distance	0.080	−0.199	0.337	0.551
**Wing length**	**0.159**	**0.070**	**0.243**	**< 0.001**

*Note:* Reference category for habitat is ‘coastal’.

**TABLE A13:** Results from the Gaussian MCMCglmm model using the reduced dataset and the alternative age. Significant predictors (i.e., their 95% CIs did not overlap with zero) are in bold.

Parameter	Estimate	Lower CI	Upper CI	pMCMC
Intercept	1.567	1.366	1.770	0.001
**Inland habitat**	**−0.523**	**−0.712**	**−0.333**	**< 0.001**
**Mixed habitat**	**−0.292**	**−0.497**	**−0.075**	**0.008**
Breeding latitude	−0.040	−0.269	0.224	0.746
Migration distance	0.084	−0.180	0.339	0.538
**Wing length**	**0.159**	**0.075**	**0.253**	**0.002**

*Note:* Reference category for habitat is ‘coastal’.

**TABLE A14:** Results from the ordinal MCMCglmm model using the reduced dataset. Significant predictors (i.e., their 95% CIs did not overlap with zero) are in bold.

Parameter	Estimate	Lower CI	Upper CI	pMCMC
Intercept	5.827	1.173	11.414	0.002
**Inland habitat**	**−529.792**	**−1045.780**	**−10.713**	**< 0.001**
**Mixed habitat**	**−4.729**	**−9.267**	**−0.824**	**0.004**
Breeding latitude	−5.442	−15.876	6.132	0.297
Migration distance	6.879924	−3.896	17.932	0.179
**Wing length**	**5.191**	**1.802**	**9.322**	**< 0.001**

*Note:* Reference category for habitat is ‘coastal’.

**TABLE A15:** Results from the ordinal MCMCglmm model using the reduced dataset and the alternative age. Significant predictors (i.e., their 95% CIs did not overlap with zero) are in bold.

Parameter	Estimate	Lower CI	Upper CI	pMCMC
Intercept	5.861	1.044	11.299	0.004
**Inland habitat**	**−365.358**	**−797.867**	**−10.160**	**< 0.001**
**Mixed habitat**	**−4.730**	**−9.363**	**−0.845**	**0.005**
Breeding latitude	−5.448	−16.278	6.430	0.310
Migration distance	6.896131	−4.580	18.407	0.206
**Wing length**	**5.120**	**1.431**	**9.295**	**< 0.001**

*Note:* Reference category for habitat is ‘coastal’.
